# Metabolic syndrome improvement in depression six months after prescribing simple hygienic-dietary recommendations

**DOI:** 10.1186/1756-0500-7-339

**Published:** 2014-06-05

**Authors:** Mauro Garcia-Toro, Margalida Gili, Olga Ibarra, Saray Monzón, Margalida Vives, Javier Garcia-Campayo, Rocío Gomez-Juanes, Miguel Roca

**Affiliations:** 1 Institut Universitari d’Investigació en Ciències de la Salut (IUNICS), University of Balearic Islands and Red de Investigación en Actividades Preventivas y Promoción de la Salud (RedIAPP), Palma, Spain; 2 Department of Psychiatry, Hospital Miguel Servet; Paseo Isabel La Catolica, 1–3; 50009 Zaragoza, Red de Investigación en Actividades Preventivas y Promoción de la Salud (RedIAPP), Palma, Spain

**Keywords:** Depression, Metabolic syndrome, Lifestyle recommendations, Diet, Exercise

## Abstract

**Background:**

Changes in diet and exercise have been separately demonstrated to improve Depression, although scientific evidence available is scarce. In a previously published controlled study, just recommending these and other lifestyle measures (sleep restriction and sunlight exposure) in combination once, patients experienced improvements in their depressive symptoms six months later. In this sample, one in three depressive patients had metabolic syndrome (MetS) at baseline. First line treatment of MetS condition is hygienic-dietetic, being Mediterranean diet and exercise especially important. Therefore we analyzed if lifestyle recommendations also improved their metabolic profile.

**Findings:**

During the sixth month evaluation, a smaller number of patients from the group receiving hygienic-dietary recommendations met MetS criteria comparing with the control group.

**Conclusions:**

This study suggests that costless lifestyle recommendations, such as exercise and Mediterranean diet, have the capacity to promote both mental and physical health in a significant proportion of depressive patients. Further research is needed to confirm or discard these preliminary findings.

## Introduction

Depression is a high prevalent disorder, prone to chronicity, and associated with a large burden in terms of suffering, disability, and healthcare costs [1,2]. One of the most common factors associated with depression is the presence of somatic disorders [3]. Metabolic syndrome (MetS) is a combination of risk factors (abdominal obesity, hypertension, dyslipidemia, and glucose intolerance) that are predictive of progression to coronary artery disease, type 2 diabetes, asthma and certain cancers [4,5]. MetS worsens with age and is rapidly growing in adults and adolescents [6,7]. The prevalence of MetS in depression vary from 25 to 41% and depression increases the risk of MetS from 1.7 to 2.8 times [8,9]. A complex bidirectional interrelationship between depression and MetSyn has been suggested [8-10]. Patients with Depression and other mental disorders may be especially predisposed to MetS since it has been proved it is triggered by psychopharmacologic drug-induced adverse effects, sedentary lifestyle, poor dietary habits, increased substance abuse and possible limited access to health care [11,12]. There are other biological mechanisms underlying the association between metabolic syndrome and depression, but are not clearly established [13]. Biological links that include increased pro-inflammatory cytokines, stress-induced hyperactivity of the hypothalamic pituitary axis (HPA), changes in homeostasis between the sympathetic and parasympathetic nervous systems and cerebral small vessel and circulation disease have been proposed [14,15,8,9]. Pathophysiology overlap of depression and MetS could offers new possibilities for simultaneous prevention and treatment. Modification of risk factors should be considered a primary target [10].

First line treatment of MetS should be based on lifestyle change, including exercise and nutrition therapy, such as Mediterranean diet [6,7,11,16]. A Mediterranean dietary pattern has been defined as comprising at least two of the following components: high monounsaturated/saturated fat ratio, low to moderate red wine consumption, high consumption of legumes, high consumption of grains and cereals, high consumption of fruits and vegetables, low consumption of meat and meat products and increased consumption of fish, and moderate consumption of milk and dairy products [17]. Diet and exercise have been suggested able to normalize immune and metabolic dysregulation in persons with MetS [18]. Diet and exercise interventions have been also demonstrated to improve depression although the evidence is still scarce [10]. However, to our knowledge, no previous research was specifically aimed to determine the impact of exercise and diet recommendations on patients with Depression and MetS.

We hypothesized that hygienic-dietary recommendations can change the metabolic profile of patients with Depression and MetSyn sixth months later.

## Methods

Eighty outpatients with a depressive episode were recruited for a randomized clinical trial that was designed to test the effectiveness of lifestyle recommendations in Depression [19,20]. The inclusion criteria were male and female patients over 18 years; experiencing a depressive episode according to DSM-IV diagnostic criteria for Major Depressive Disorder, Dysthymic Disorder or Bipolar Disorder; receiving antidepressant treatment; patients who have the ability to communicate in Spanish and give informed written consent and women of childbearing age practicing safe contraceptive methods. Exclusion criteria were: any other severe disease that affects the CNS; uncontrolled or potentially interfering medical condition; delusions or hallucinations at the time of the study; significant risk of suicide and pregnancy or lactation.

Complete analytic parameters (basal and six month’s data) were available for fifty-one of those patients who finished the study. Reasons for non-completion included patient decision, protocol violation or appearance of a severe, uncontrolled, or potentially interfering medical condition. The study protocol was approved by the local ethical board (CEIC: Comité Ético y de Investigación Clínica de las Islas Baleares), and was registered (Trial registration: Current Controlled Trials ISRCTN59506583) and published elsewhere [19].

After signed informed consents outpatients were randomly assigned either to the active treatment or the control group. Patients assigned to the active treatment group received an envelope containing a sheet of paper with the four hygienic-dietary recommendations under consideration. Two of them suggested diet changes, based on Mediterranean diet guidelines, and exercising daily:

1. *Go to bed when sleepy and not before 11 p.m. Use your bed and bedroom only for sleep and sex (do not read, watch TV or lie on the bed during the day). If you do not fall asleep after 15 or 20 minutes, get up and start an activity until you feel sleepy enough to go back to bed. Get up early, never later than 9 a.m., no matter how well you have slept the night before. Do not lie down or take a nap during the day.*

2. *Walk at least 1 hour a day, at a good pace but without becoming short of breath or being unable to talk while walking. If you think you have a medical problem which makes walking difficult or uncomfortable consult your doctor. Use appropriate footwear for walking and have a shower or a bath afterwards.*

3. *Expose yourself to sunlight at least 2 hours per day, taking precautions to avoid sunburn or sunstroke (sunscreen, hat, etc.).*

4. *Try to eat a healthy and balanced diet. Eat at regular hours without snacking between meals. Avoid especially sweet or sugary drinks. Eat fish at least three times per week, plus fruit, cereals, nuts and vegetables daily.*

The control group received an identical envelope, but in this case the recommendation was to perform the pattern of eating and exercise according to what they thought might make them feel better:

1. *Sleep the hours that you feel your body needs.*

2. *Adapt the pace of daily physical activity that meets your needs.*

3. *If exposed to sunlight take precautions to avoid sunburn or sunstroke (sunscreen, hat, etc.).*

4. *Try to eat a healthy and balanced diet.*

The Mini International Neuropsychiatric Interview (MINI), the Hamilton rating scale for depression (HAM-D) 17-item version, the 21-item Beck Depression Inventory, and the Clinical Global Impression scale (CGI) were used. After the sixth month intervention tests were again administered by blind raters. During this time the patients were taking antidepressants, and continued to be treated by their GP or psychiatrist without any interference from the research team. Analytical determinations, blood pressure and weight were measured at baseline and after six months. The “International Diabetes Federation” (IDF) criteria were used to define MetS in the patients [21]: They must have central obesity, defined as excessive waist circumference, with sex and ethnic specific values (≥94 cm for European men) or BMI > 30 kg/m^2^ plus two or more of the following four factors; raised triglycerides level: ≥ 150 mg/dl or specific treatment for this lipid abnormality; reduced concentration of high density lipoprotein (HDL): < 40 mg/dl in men and < 50 mg/dl in women or specific treatment for this lipid abnormality; raised blood pressure: systolic blood pressure ≥ 130 mmHg or diastolic blood pressure ≥ 85 mmHg or treatment of previously diagnosed hypertension; raised fasting plasma glucose concentration ≥ 100 mg/dl or previously diagnosed type 2 diabetes.

We conducted a statistical analysis using Fisher’s exact test for categorical variables and U Man-Whitney test for continuous variables.

## Findings

Main socio-demographic and clinical data are presented in Table 1. The antidepressants prescribed were very similar in both groups; most of the patients used dual and selective serotonin reuptake inhibitors. Scales indicated an improvement of depressive symptoms in the active treatment group, but due to the insufficient sample not all the items reached statistical significance. Adverse effects resulting from these recommendations did not appear. Concerning to the metabolic variables shown in Table 1, most changes were in terms of improving the metabolic profile in the active group versus control recommendations. There was a statistically significant change between pre and post condition in the number of patients in both groups that met MetS criteria (X2 = 4.69, p = 0.03).

**Table 1:** Demographic and clinical results

	***Active n = 25***	***Control n = 26***	***P value***
Female sex, N°	20	17	0.24
Age y, Mean (SD)	47.6 (11.6)	50.6 (11.4)	0.36
Major Depression, N°	23	20	0.28
HAM-D increment	- 8.80 (8.9)	- 5.57 (4.9)	0.11
BDI increment	- 8.64 (9.2)	- 4.25 (6.3)	0.05
GCI increment	- 1.72 (1.3)	- 0.69 (1.0)	0.00
BMI increment	- 0.11 (1.5)	0.29 (1.6)	0.46
SBP increment	- 2.33 (15.7)	- 2.04 (16.9)	0.95
DBP increment	1.5 (12.9)	- 1.76 (15.0)	0.47
Serum glucose increment	- 3.9 (20.7)	- 0.6 (21.3)	0.58
Total Cholesterol increment	- 3.92 (21.9)	3.76 (39.2)	0.39
LDL Cholesterol increment	- 7.00 (19.5)	3.00 (33.14)	0.20
HDL Cholesterol increment	1.76 (8.3)	- 0.15 (8.1)	0.40
Triglycerides increment	6.4 (41.9)	9.3(62.8)	0.84
Metabolic Syndrome pre, N°	7	9	
Metabolic Syndrome post, N°	3	10	0.03

Hamilton 17-item scale (HAM-D), Beck Depression Inventory 21-item (BDI, Global Clinical Impression scale (GCI), Body Mass Index (BMI), Systolic Blood Pressure (SBP), Diastolic Blood Pressure (DBP).

## Discussion

The main finding of this study is that MetS was lower in those patients receiving the active hygienic-dietary recommendations compared to the control group. Perhaps due to lack of statistical power, our study has not found differences among any of the variables included in MetS definition. Nevertheless, it has been suggested that it is more important the association of Depression with the construct of MetS rather than with its components [10].

Seasonality of serum lipids and weight/body mass index may affect MetS prevalence raising their development in the winter [22]. Changes of plasma volume linked to seasonal variations in temperature and/or physical activity could be responsible of this fact [22]. However, these findings cannot explain our results, since patients were assessed throughout the year and assigned randomly to both groups.

Depressed people are more prone to eat highly palatable and caloric food, rich in cholesterol and carbohydrate and low in fish, vegetable and cereals. This diet does not ensure an adequate intake of B vitamins, folic acids, and essential fatty acids for vascular, inflammatory and metabolic balance, and may induce MetS [10]. Depression, when in comorbidity with MetS, shows worse course, more severe clinical picture and a higher tendency to chronicity and suicidality besides lower social functioning [10]. It has been proposed the concept of “metabolic depression” to identify a chronic subtype of depression more frequent in late-life, with metabolic abnormalities and a protracted course [10,21]. Our preliminary data could indicate that this situation can be faced better when combining antidepressant treatment with dietary hygienic measures.

Our study has some weaknesses that need to be acknowledged for proper interpretation. The main limitations of this study are the small sample size and the short follow-up period. However, the small sample size would not explain our positive findings but makes the type I statistical error more probable. Although the vast majority of patients met criteria for Major Depression, we also had patients with Dysthymic and Bipolar Disorder. Due to the limited sample we could not carry out a separate analysis of these patients to determine if they have the same behavior against lifestyle recommendations.

Depression has been associated with poor adherence to diet and exercise regimens [14,18]. There is no measure of adherence in this study. This is a clear limitation because low adherence to the study recommendations cannot be ruled out and this reinforces the fact that these findings may simply be chance. Nevertheless we demonstrated in a pilot study with a small part of these patients using actigraphs, that they did change their lifestyle habits, at least in a short-term period [23]. It would have been interesting to dispose of objective adherence measures from all the patients during the follow-up. Unfortunately, it could not be put into practice due to logistical limitations.

The benefits of lifestyle changes for patients experiencing a depressive episode and Metabolic Syndrome could be achieved by using a simple combination of hygienic-dietary recommendations on a written piece of paper. These benefits applied to metabolic profile are a key factor for patients’ future cardiovascular health which also constitutes a key factor for their Mental Health evolution [1,24-26]. The improvement of the metabolic profile found in this study would be an extra-motivation for patients in order to maintain these lifestyle changes in the long-term [16].

## Competing interests

The authors declare they have no financial or non-financial competing interests in relation to this manuscript.

## Authors’ contributions

OI, MG, MR, MG and JG were substantially involved in the conception of the study and participate in its design. OI, MG, MR, SM, MV and MS participated in the coordination of the study and the acquisition of data. MG drafted the manuscript with the assistance of RG, SM, OI, MR, MG and JG. The other authors critically revised the text. All have approved the present publication.

## References

[B1] Gutierrez-Fraile M Garcia-Calvo C Prieto R Gutierrez-Garitano I Mental disorders in psychiatric outpatients in spain Actas Esp Psiquiatr 2011 39 349 355 22127907

[B2] Rubio JM Markowitz JC Alegria A Pérez-Fuentes G Liu SM Lin KH Blanco C Epidemiology of chronic and nonchronic major depressive disorder: results from the national epidemiologic survey on alcohol and related conditions Depress Anxiety 2011 28 622 631 10.1002/da.20864 21796739 PMC3212845

[B3] Garcia-Toro M Rubio JM Gili M Roca M Jin CJ Liu SM Bastianoni C Blanco C Persistence of chronic major depression: a national prospective study J Affect Disord 2013 151 306 312 10.1016/j.jad.2013.06.013 23866303

[B4] Kahl KG Greggersen W Schweiger U Cordes J Balijepalli C Lösch C Moebus S Prevalence of the metabolic syndrome in unipolar major depression Eur Arch Psychiatry Clin Neurosci 2012 262 313 320 10.1007/s00406-011-0277-4 22183567

[B5] Jin H Lanouette NM Mudaliar S Henry R Folsom DP Khandrika S Glorioso DK Jeste DV Association of posttraumatic stress disorder with increased prevalence of metabolic syndrome J Clin Psychopharmacol 2009 29 210 215 10.1097/JCP.0b013e3181a45ed0 19440072 PMC3640506

[B6] Rubenfire M Mollo L Krishnan S Finkel S Weintraub M Gracik T Kohn D Oral EA The metabolic fitness program: lifestyle modification for the metabolic syndrome using the resources of cardiac rehabilitation J Cardiopulm Rehabil Prev 2011 31 282 289 10.1097/HCR.0b013e318220a7eb 21734589

[B7] Garcia-Toro M Roca M Monzon S Vives M Oliván B Vicens E Salva J Gili M Hygienic-dietary recommendations for major depression treatment: study protocol of a randomized controlled trial BMC Psychiatry 2012 12 201 10.1186/1471-244X-12-201 23158080 PMC3528444

[B8] Suttajit S Pilakanta S Prevalence of metabolic syndrome and its association with depression in patients with schizophrenia Neuropsychiatr Dis Treat 2013 9 941 946 23882141 10.2147/NDT.S47450 PMC3709830

[B9] Grover S Nebhinani N Chakrabarti S Avasthi A Kulhara P Metabolic syndrome in drug-naive patients with depressive disorders Indian J Psychol Med 2013 35 167 173 10.4103/0253-7176.116247 24049228 PMC3775049

[B10] Marazziti D Rutigliano G Baroni S Landi P Dell'osso L Metabolic syndrome and major depression CNS Spectr 2013 8 1 12 24103843 10.1017/S1092852913000667

[B11] Pan A Keum N Okereke OI Sun Q Kivimaki M Rubin RR Hu FB Bidirectional association between depression and metabolic syndrome: a systematic review and meta-analysis of epidemiological studies Diabetes Care 2012 35 1171 1180 10.2337/dc11-2055 22517938 PMC3329841

[B12] Hidaka BH Depression as a disease of modernity: explanations for increasing prevalence J Affect Disord 2012 140 205 214 10.1016/j.jad.2011.12.036 22244375 PMC3330161

[B13] Hamer M Batty GD Kivimaki M Risk of future depression in people who are obese but metabolically healthy: the English longitudinal study of ageing Mol Psychiatry 2012 17 940 945 10.1038/mp.2012.30 22525487 PMC3428506

[B14] Alvarez A Faccioli J Guinzbourg M Castex MM Bayón C Masson W Bluro I Kozak A Sorroche P Capurro L Grosembacher L Proietti A Finkelsztein C Costa L Fainstein Day P Cagide A Litwak LE Golden SH Endocrine and inflammatory profiles in type 2 diabetic patients with and without major depressive disorder BMC Res Notes 2013 6 61 10.1186/1756-0500-6-61 23410093 PMC3599430

[B15] Sekita A Arima H Ninomiya T Ohara T Doi Y Hirakawa Y Fukuhara M Hata J Yonemoto K Ga Y Kitazono T Kanba S Kiyohara Y Elevated depressive symptoms in metabolic syndrome in a general population of Japanese men: a cross-sectional study BMC Public Health 2013 13 862 10.1186/1471-2458-13-862 24044502 PMC3848461

[B16] Toobert DJ Glasgow RE Strycker LA Barrera M Jr Ritzwoller DP Weidner G Long-term effects of the Mediterranean lifestyle program: a randomized clinical trial for postmenopausal women with type 2 diabetes Int J Behav Nutr Phys Act 2007 4 1 10.1186/1479-5868-4-1 17229325 PMC1783667

[B17] Rees K Hartley L Flowers N Clarke A Hooper L Thorogood M Stranges S 'Mediterranean' dietary pattern for the primary prevention of cardiovascular disease Cochrane Database Syst Rev 2013 8 CD009825 23939686 10.1002/14651858.CD009825.pub2

[B18] Penninx BW Milaneschi Y Lamers F Vogelzangs N Understanding the somatic consequences of depression: biological mechanisms and the role of depression symptom profile BMC Med 2013 11 129 10.1186/1741-7015-11-129 23672628 PMC3661358

[B19] Garcia-Toro M Ibarra O Gili M Salva J Monzón S Vives M Serrano MJ Garcia-Campayo J Roca M Effectiveness of hygienic-dietary recommendations as enhancers of antidepressant treatment in patients with depression: study protocol of a randomized controlled trial BMC Public Health 2010 10 404 10.1186/1471-2458-10-404 20618920 PMC2910684

[B20] Garcia-Toro M Ibarra O Gili M Serrano MJ Oliván B Vicens E Roca M Four hygienic-dietary recommendations as add-on treatment in depression: a randomized-controlled trial J Affect Disord 2012 140 200 203 10.1016/j.jad.2012.03.031 22516309

[B21] Marijnissen RM Smits JE Schoevers RA van den Brink RH Holewijn S Franke B de Graaf J Oude Voshaar RC Association between metabolic syndrome and depressive symptom profiles-sex-specific? J Affect Disord 2013 151 1138 1142 10.1016/j.jad.2013.07.029 24011730

[B22] Altinbas K Guloksuz S Oral ET Metabolic syndrome prevalence in different affective temperament profiles in bipolar-I disorder Rev Bras Psiquiatr 2013 35 131 135 10.1590/1516-4446-2011-0746 23904017

[B23] García-Toro M Ibarra O Gili M Serrano MJ Vives M Monzón S Bauzá N Oliván B Vicens E Roca M Adherence to lifestyle recommendations by patients with depression Rev Psiquiatr Salud Ment 2012 5 236 40 10.1016/j.rpsm.2012.04.003 23021296

[B24] Gans RO The metabolic syndrome, depression, and cardiovascular disease: Interrelated conditions that share pathophysiologic mechanisms Med Clin North Am 2006 90 573 591 10.1016/j.mcna.2006.05.002 16843763

[B25] Sanchez-Villegas A Delgado-Rodriguez M Alonso A Schlatter J Lahortiga F Serra Majem L Martínez-González MA Association of the mediterranean dietary pattern with the incidence of depression: The seguimiento universidad de Navarra/University of navarra follow-up (SUN) cohort Arch Gen Psychiatry 2009 66 1090 1098 10.1001/archgenpsychiatry.2009.129 19805699

[B26] Salvi V Albert U Chiarle A Soreca I Bogetto F Maina G Metabolic syndrome in italian patients with bipolar disorder Gen Hosp Psychiatry 2008 30 318 323 10.1016/j.genhosppsych.2008.04.009 18585534

